# Superior extraconal orbital fat hyperintensityin pediatric population: a potential diagnostic pitfall

**DOI:** 10.1007/s00247-026-06543-z

**Published:** 2026-02-07

**Authors:** Ariel Kerpel, Tamer Sobeh, Eyal Atia, Israel Cohen, Chen Hoffmann, Shai Shrot

**Affiliations:** 1 Sheba Medical Center, 2nd Sheba Rd, Ramat Gan, 52621 Israel; 2 Gray Faculty of Medical and Health Sciences, Tel Aviv University, Tel Aviv, Israel

**Keywords:** Child, Finding, incidental, Magnetic resonance imaging, Misdiagnosis, Orbit

## Abstract

**Background:**

With the increasing use of magnetic resonance imaging (MRI) in children, radiologists frequently encounter incidental findings that may mimic pathology. One such underrecognized finding is T2-weighted hyperintensity in the superior extraconal orbital fat, which is occasionally mistaken for an infiltrative or neoplastic process.

**Objective:**

Our objective was to characterize the imaging appearance, prevalence, and clinical associations of superior extraconal orbital fat T2 hyperintensity in pediatric MRI.

**Materials and methods:**

We conducted a retrospective study of 143 pediatric patients (mean age 7.2±5.1 years) who underwent brain MRI with an orbit-specific protocol between 2015 and 2022. Patients were grouped based on the presence or absence of bilateral papilledema and whether imaging was performed under general anesthesia. Clinical data were extracted from the electronic medical records. Three neuroradiologists reviewed images for the presence of a hyperintense signal along the superior extraconal orbital fat. Interobserver agreement was calculated using Fleiss’ kappa. Univariate and multivariable logistic regression analyses were performed to assess associations with age, anesthesia, gender, and magnet strength.

**Results:**

Symmetric T2-hyperintense bands along the superior extraconal orbital fat were observed in 45.5% of patients. The finding was more common in younger children (4.4±3.9 years vs. 9.6±4.8 years; *P*<0.001). Multivariate analysis showed a significant negative correlation with age (*P*<0.001) and a positive correlation with papilledema (*P*=0.012), but no independent association with gender, anesthesia, or magnet strength. The hyperintensity was non-enhancing or only subtly enhancing. Clinical follow-up demonstrated no subsequent orbital or infiltrative pathology in the majority of patients, and most patients without documented follow-up underwent ambulatory MRI for evaluation of strabismus, which showed no evidence of infiltrative or other orbital disease.

**Conclusion:**

Superior extraconal orbital fat T2 hyperintensity is a relatively common, likely non-pathologic MRI finding in pediatric patients, particularly in younger children, and the apparent association with anesthesia likely reflects age-related confounding. Awareness of this benign appearance may help avoid diagnostic confusion and prevent unnecessary workup or intervention.

**Graphical Abstract:**

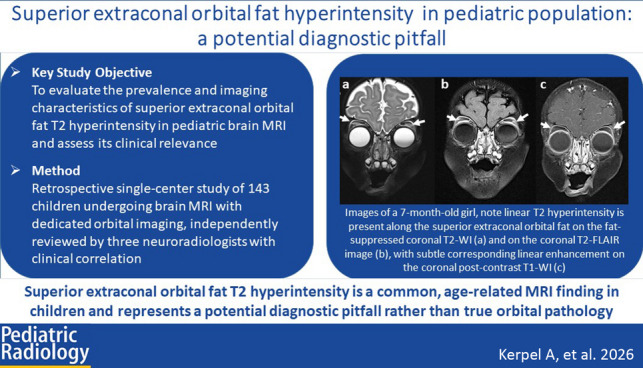

## Introduction

Magnetic resonance imaging (MRI) is increasingly utilized in the pediatric population, offering high-resolution, radiation-free assessment of intracranial and orbital structures. With the broader adoption of high-field-strength scanners and advanced imaging protocols, radiologists are frequently confronted with incidental imaging findings, features unrelated to the clinical indication, that may mimic pathology and pose diagnostic challenges [1].

One such underrecognized observation is T2-weighted hyperintensity localized along the superior extraconal orbital fat, a finding most commonly seen in young children undergoing dedicated MRI of the orbit. Although often subtle and symmetric, this signal abnormality may appear asymmetric or exhibit mild post-contrast enhancement. Such appearances can simulate orbital edema, infiltrative lesions, or neoplastic processes, raising concern for true pathology in otherwise asymptomatic patients. Despite its characteristic imaging pattern and apparent frequency, this finding has not been systematically described in the neuroradiological literature. Its potential to trigger unnecessary diagnostic workup underscores the need for increased awareness and clarification of its clinical relevance. This phenomenon may resemble other well-documented variants such as torcular pseudomass or benign cystic structures (e.g., choroid plexus or pineal cysts), which are now recognized as non-pathologic entities with no clinical consequence [2, 3].

This study aimed to evaluate the prevalence, imaging characteristics, and clinical correlates of superior extraconal orbital fat T2 hyperintensity in a pediatric cohort. Specifically, we investigated its associations with demographic and clinical features. A clearer understanding of this entity may help radiologists avoid diagnostic pitfalls and reduce unnecessary workup in the pediatric population.

## Methods

### Study design and patient selection

This retrospective study was approved by the institutional review board, with a waiver of informed consent. Pediatric patients (under 18 years of age) who underwent brain MRI with an orbit-specific protocol between 2015 and 2022 were identified through a computerized query of the institutional Radiology Information System (Carestream Vue RIS). Patients were categorized by papilledema status and anesthesia status. The presence of papilledema was confirmed by ophthalmologic diagnosis of bilateral papilledema, as determined by ophthalmologic fundoscopy. Bilateral papilledema was selected as a representative clinical sign of elevated intracranial pressure. Non-papilledema patients underwent orbit MRI for the evaluation of strabismus, diplopia, or blurred vision without papilledema. Exclusion criteria included absent or technically suboptimal volumetric T2 orbital sequences, underlying orbital structural abnormalities, and unilateral papilledema. The presence of an endotracheal tube or a laryngeal mask, visible on sagittal T1-weighted images, determined anesthesia status. General anesthesia at our institution typically involves induction with propofol followed by maintenance with sevoflurane inhalation. Accordingly, the study cohort was categorized into sedated and non-sedated groups, with both groups including patients with and without papilledema.

### Imaging protocol

MRI examinations were performed using either 1.5-tesla (T) or 3-T systems from multiple vendors (GE Healthcare, Madison, WI; Philips Medical Systems, Eindhoven, Netherlands; and Siemens Healthcare, Erlangen, Germany). Standard clinical brain imaging sequences included pre- and post-contrast T1-weighted images, T2-weighted images, T2-fluid-attenuated inversion recovery (FLAIR), diffusion-weighted imaging (DWI), and blood-sensitive sequences (T2* gradient-echo or susceptibility-weighted imaging). Dedicated orbital imaging was performed using axial and coronal 2–3 mm T2-weighted and post-contrast T1-weighted images, all acquired with fat saturation. All patients underwent submillimeter isotropic high-resolution volumetric T2-weighted imaging, e.g., fast imaging employing steady-state acquisition (FIESTA), constructive interference in steady state (CISS), sampling perfection with application-optimized contrasts using different flip-angle evolutions (SPACE), and turbo spin echo (TSE), with matrix dimensions exceeding 340×340 and voxel sizes ranging from 0.75 mm^3^ to 0.9 mm^3^.

### Image analysis and interobserver variability

For each patient, the presence of T2 signal abnormality was assessed on coronal fat-suppressed T2-weighted images at the level of the distal optic nerve and proximal globe (to avoid the upper eyelid tissue). In cases in which diagnostic coronal fat-suppressed T2-weighted images were unavailable, coronal fat-suppressed T2-FLAIR images were evaluated. Hyperintensity was defined as increased T2 signal along the superior orbital roof, i.e., in the extraconal orbital fat, compared to other orbital walls. Post-contrast enhancement was evaluated using coronal fat-suppressed post-contrast T1-weighted images. Image interpretation was independently performed by three radiologists: a neuroradiology fellow (E.A.) and two board-certified neuroradiologists (T.S. and A.K., with 1 year and 2 years of post-certification experience, respectively); all readers were blinded to the patients’ clinical and demographic data. In cases of disagreement among the three readers, consensus was achieved following review by a senior pediatric neuroradiologist (S.S., 9 years of experience). Interobserver agreement was quantified using Fleiss’ kappa statistic. Values between 0.41 and 0.60 represent moderate agreement, while values between 0.61 and 0.80 indicate substantial agreement.

### Clinical follow-up

Clinical follow-up was performed through a review of the electronic medical records by the senior author (S.S.). Baseline comorbidities and the subsequent development of medical conditions potentially associated with orbital infiltration were recorded. Follow-up data included documented diagnoses, clinical evaluations, and relevant imaging findings, when available.

### Statistical analysis

All statistical analyses were conducted using SPSS software (version 29.0; IBM Corp., Armonk, NY). Descriptive statistics were reported as mean, standard deviation, and range for continuous variables, and as counts and percentages for categorical variables. Univariate analysis was first conducted to compare demographic and clinical characteristics between patients with and without superior extraconal orbital fat T2 hyperintensity and enhancement. The Mann–Whitney *U* test was used for continuous variables, and the chi-square test was applied for categorical variables. Various variables were entered into a multivariable binary logistic regression model to identify independent predictors of T2 signal abnormality. Statistical significance was set at *P*<0.05.

## Results

### Study cohort

A total of 143 pediatric patients (mean age 7.2±5.1 years) were included in the study. The cohort consisted of 66 females (46.2%). Papilledema was diagnosed in 44 patients (30.8%), while 99 patients (69.2%) did not have papilledema. MRI was performed under general anesthesia in 91 cases (63.6%), while 52 patients (36.4%) underwent imaging without anesthesia. The majority of scans (134/143, 93.7%) were acquired on 3-T scanners, with only 6.3% performed on 1.5-T scanners. Table 1 summarizes demographic and clinical data.

**Table 1:** Demographic characteristics of the study cohort

	No anesthesia		With anesthesia		Total	*P*-value^a^
	No papilledema	Papilledema	**N**o papilledema	Papilledema		
*n*	28	24	71	20	143	
Age (years)	11.9±3.3	13±3.2	3.8±3.1	6.1±3.4	7.2±5.1	<0.001
Male (%)	14/28	9/24	39/71	15/20	77/143	0.08
3-T scanner (%)	25/28	20/24	69/71	20/20	134/143	0.008

^a^Comparison between anesthesia and non-anesthesia groups

### Imaging findings

**Fig. 1:**
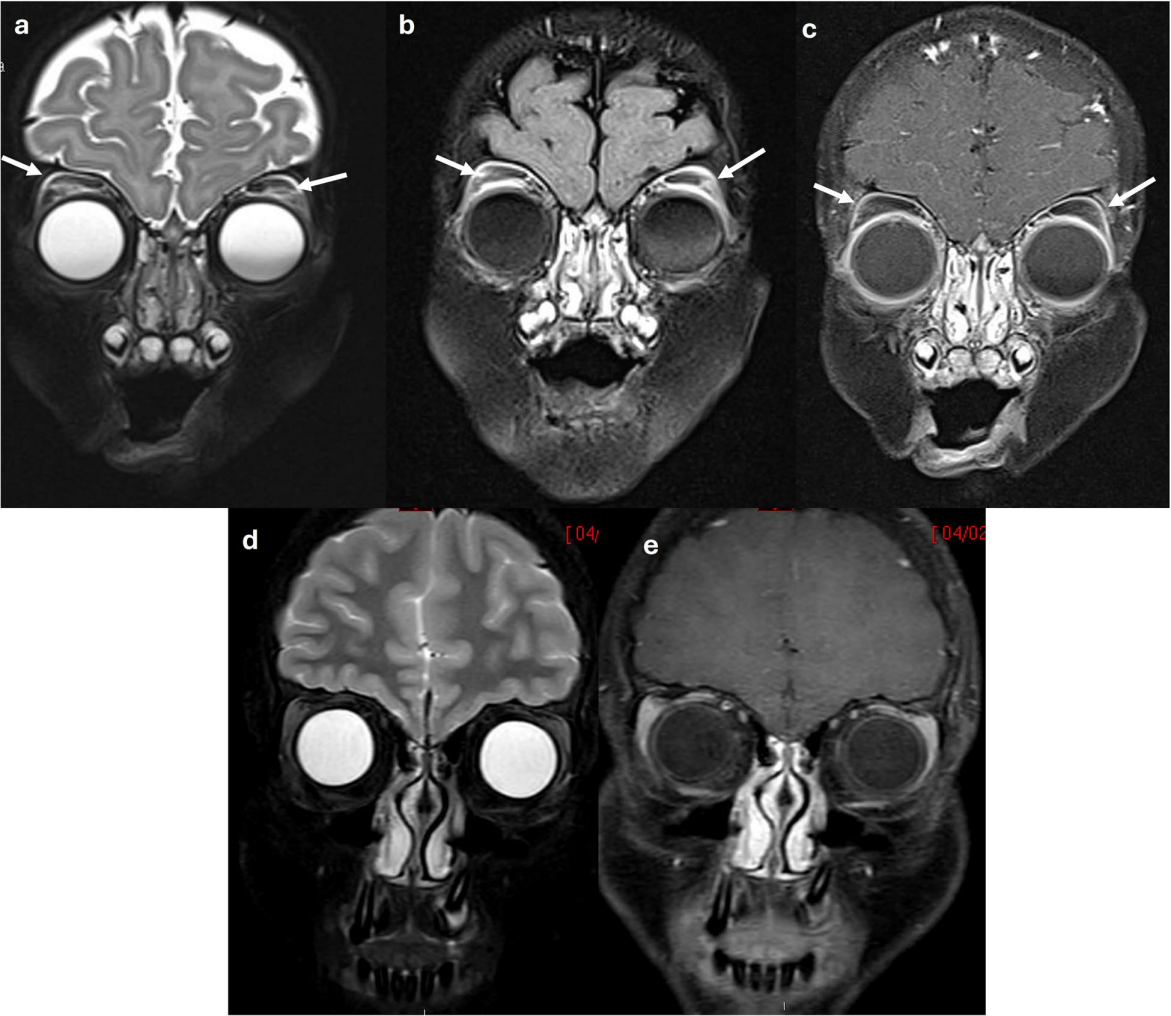
Images of a 7-month-old girl (**a**–**c**) and a 13-year-old girl (**d**–**e**). In the younger patient, linear T2 hyperintensity is present along the superior extraconal orbital fat on the fat-suppressed coronal T2-weighted image (**a**, *arrows*) and on the coronal T2-FLAIR image (**b**, *arrows*), with subtle corresponding linear enhancement on the coronal post-contrast T1-weighted image (**c**, *arrows*). The older patient demonstrates normal signal intensity of the superior extraconal orbital fat on the fat-suppressed coronal T2-weighted image (**d**) and on the coronal post-contrast T1-weighted image (**e**)

T2 hyperintensity in the superior extraconal orbital fat was observed in 45.5% of patients, while T1 enhancement was identified in 7.7% of cases (Fig. 1, Table 2).

**Table 2:** Imaging findings in superior extraconal orbital fat

	No anesthesia		With anesthesia		Total	*P*-value^a^	*P*-value^b^	*P*-value^c^	*P*-value^d^
	No papilledema	Papilledema	No papilledema	Papilledema					
*n*	28	26	71	20	143				
T2 hyperintensity	1 (3.6%)	8 (33.3%)	44 (62%)	12 (60%)	65 (45.5%)	<0.001	1.0	<0.001	0.077
T1 enhancement^e^	1 (3.6%)	2 (8.3%)	7 (9.9%)	2 (10.0%)	11 (7.7%)^e^	0.206^e^	0.737	0.185	1.0

^a^Comparison between patients with and without anesthesia

^b^Comparison between patients with and without papilledema

^c^Comparison between anesthesia and non-anesthesia subgroups of patients without papilledema

^d^Comparison between anesthesia and non-anesthesia subgroups of patients with papilledema

^e^Based on 140 cases (3 cases with missing T1 data)

Interobserver reliability was assessed using Fleiss’ kappa for both T2 hyperintensity and post-contrast T1 enhancement at the superior extraconal orbital fat. For T2 signal evaluation, overall agreement among the three raters was moderate, with a Fleiss’ kappa of 0.42 (95% confidence interval (CI), 0.33–0.52; *P*<0.001). Agreement was higher for the absence of T2 hyperintensity (conditional agreement=0.785) compared to its presence (conditional agreement=0.639). For T1 contrast enhancement, overall agreement was lower, with a Fleiss’ kappa of 0.353 (95% CI, 0.258–0.449; *P*<0.001). Agreement was notably higher for negative cases (no enhancement; conditional agreement=0.933) than for positive cases (conditional agreement=0.422). Inter-reader assessment demonstrated high overall symmetry of the orbital T2-hyperintensity, with symmetry proportions ranging from 73.8% to 98.5%. 

We performed univariate analyses to identify factors associated with increased T2 hyperintensity of superior extraconal orbital fat. Age was also significantly associated with T2 signal abnormalities. A Mann-Whitney *U* test demonstrated that patients with increased T2 signal were significantly younger than those without (mean age, 4.4±3.9 years vs. 9.6±4.8 years; *P*<0.001). In addition, imaging on a 3-T MRI scanner was more frequently associated with increased T2 signal than that on 1.5-T scanners (*P*=0.04). Children who underwent MRI under anesthesia demonstrated a markedly higher prevalence of T2 hyperintensity compared to those scanned without anesthesia (*P*<0.001). No significant associations were found between increased T2 signal and gender (*P*=0.178), or the presence of papilledema (*P*=1).

A multivariate binary logistic regression analysis was performed to evaluate the independent associations of age, papilledema, anesthesia status, sex, and MRI field strength with T2 hyperintensity. Increasing age was independently associated with a lower likelihood of T2 hyperintensity (*P*<0.001), indicating a strong inverse relationship that persisted after adjustment for all covariates. After accounting for age and the other variables in the model, papilledema emerged as an independent predictor, conferring a significantly increased likelihood of T2 hyperintensity (*P*=0.012). In contrast, anesthesia status, sex, and magnet strength were not significantly associated with the orbital T2 hyperintensity in the adjusted model (all *P*>0.6). Although univariate analyses did not show an association, the multivariate model revealed that age and papilledema have independent and opposite effects, with the impact of papilledema becoming apparent only after adjusting for other parameters, which serve as major confounders.

### Clinical follow-up

Clinical follow-up data were available for 43 of the 65 patients with superior extraconal orbital fat T2 hyperintensity. The median duration of clinical follow-up was 30 months (IQR, 13–41.5 months). Among the 22 patients without documented clinical follow-up, 17 were referred for ambulatory evaluation of strabismus; none demonstrated papilledema. One patient underwent evaluation for an epidermoid cyst without evidence of papilledema. One patient was later diagnosed with idiopathic intracranial hypertension and presented with papilledema. Another patient presented with a headache without papilledema. In two additional cases, papilledema was suspected at presentation, but no clinical follow-up data were available.

## Discussion

In this retrospective study, we found that approximately 45% of our pediatric cohort exhibited T2 hyperintensity in the superior extraconal orbital fat, a finding that was more prevalent in younger children. The appearance was more commonly observed in patients with papilledema, a clinical marker of elevated intracranial pressure. Univariate analysis showed it was also more common in scans performed under anesthesia and using 3-T MRI systems, though these findings were probably confounded by factors such as age (as demonstrated in multivariate analysis). No association was identified with gender. Clinical follow-up, especially in patients with papilledema, supports the interpretation of this superior extraconal orbital fat T2 hyperintensity as a benign imaging finding. While the finding was inconsistently reported in radiologic interpretations, in some cases, it was sufficiently pronounced to raise concern for an infiltrative or mass-like process and to prompt clinical attention or follow-up imaging, especially if not recognized as a benign finding.

Given its imaging appearance, typically symmetric, curvilinear, and more pronounced in younger children, we hypothesize that it may represent normal lymphatic tissue or loose connective tissue along the superior extraconal orbital fat, which is more prominent in younger children and might become less conspicuous with age. One speculative possibility is that this signal reflects the presence of lymphatic tissue along the superior extraconal orbital fat. This hypothesis draws a partial analogy from recent imaging studies of the dural meningeal lymphatic system. Lymphatic vessels have been histologically and radiologically identified along the superior sagittal sinus, where they can occasionally be visualized as linear structures on contrast-enhanced T1-weighted or T2-FLAIR MRI sequences [4, 5]. These meningeal lymphatics tend to be more prominent in younger individuals and progressively diminish with age. It is conceivable that a similar lymphatic or perilymphatic structure exists in the superior orbit and contributes to the observed increase in T2 signal. Another possible explanation is differences in tissue composition between children and adults, such as variations in the amounts of fat and water in their soft tissues [6, 7]. Compared to adults, the immature composition of periorbital fat in children is characterized by higher water content, increased cellularity, and increased vascularity. These developmental features contribute to greater MRI signal-intensity variability, particularly on T2-weighted sequences, where fat may exhibit heterogeneous signal patterns, including subtle hyperintensity. These signal-intensity variations are more pronounced in younger children as periorbital fat continues to mature.

Multivariate analysis found that papilledema was independently associated with superior extraconal orbital fat T2 hyperintensity. This might reflect a pressure-related disturbance of perioptic and periorbital fluid homeostasis. Experimental and anatomic studies support CSF outflow and tracer movement along perineural routes and meningeal lymphatic pathways that can communicate with periorbital lymphatics and interstitial tissue, providing a potential mechanism whereby elevated intracranial pressure could increase subtle interstitial fluid accumulation in the superior extraconal region [8, 9].

When encountering increased T2 signal along the superior extraconal orbital fat in pediatric brain MRI, a focused differential diagnosis should include neoplastic, infiltrative, inflammatory, and infectious processes. While entities such as rhabdomyosarcoma and Langerhans cell histiocytosis (LCH) may involve the superior orbit, they typically appear as unilateral, enhancing soft-tissue masses with displacement of adjacent structures or osseous erosion, features not observed in our cohort [10]. Similarly, leukemic infiltration tends to present as diffuse orbital thickening, sometimes mimicking inflammation, but is usually associated with more extensive soft-tissue involvement. Metastatic neuroblastoma, known for involving the orbital roof, often demonstrates bone destruction and enhancing extraconal masses, frequently with clinical signs such as periorbital ecchymosis. Inflammatory and infectious conditions, such as orbital pseudotumor or cellulitis, typically exhibit prominent enhancement, involvement of multiple orbital compartments, and are often clinically symptomatic [10, 11]. Although the exact nature of orbital T2 hyperintensity remains unclear, its strong association with young age and lack of correlation with clinical symptoms suggest that it may represent a benign, non-pathologic imaging finding. In the absence of other worrisome orbital imaging features, such as solid mass, bony changes, and mass effect, or clinical suspicion, we believe this finding should be recognized as a potential imaging pitfall rather than a sign of true pathology.

Importantly, the T2 hyperintensity in the extraconal fat was consistently observed despite uniform orbital fat saturation, making a fat-saturation artifact unlikely. Failure or inhomogeneity of fat suppression is known to produce misleading signal alterations that may falsely suggest edematous or infiltrative changes within the orbit [12]. In our study, homogeneous suppression of the surrounding orbital fat supports the interpretation of a true imaging finding rather than a technical artifact.

Our study has several limitations. Its retrospective nature introduces potential selection bias and limits uniformity in imaging protocols and clinical data. Consequently, group sizes were unequal, reflecting the heterogeneity of clinical indications and patient populations encountered in routine practice. Differences in scanner models and sequence parameters may have influenced the visibility and characterization of the signal abnormality. The intensity and availability of clinical follow-up varied according to the initial clinical presentation, potentially introducing verification bias, although no subsequent orbital or infiltrative pathology was identified in patients with available follow-up. Although the T2 hyperintense signal was consistently observed, the absence of histopathologic correlation limits definitive interpretation of its underlying nature.

In conclusion, we describe a distinct, symmetric T2 hyperintense signal along the superior extraconal orbital fat in pediatric brain MRI, most commonly observed in younger children. This imaging finding shows no or only subtle enhancement, lacking associated mass effect or osseous change. While the precise etiology remains uncertain, the imaging and clinical characteristics support a benign, likely non-pathologic condition. Recognition of this pattern may help avoid misinterpretation and unnecessary follow-up in clinical practice.

## Data Availability

No datasets were generated or analysed during the current study.
